# Sign Reversal of Spin‐Transfer Torques

**DOI:** 10.1002/advs.202309467

**Published:** 2024-04-16

**Authors:** Dae‐Yun Kim, Qurat ul Ain, Yune‐Seok Nam, Ji‐Sung Yu, Seong‐Hyub Lee, Jun‐Young Chang, Kitae Kim, Woo‐Young Shim, Duck‐Ho Kim, Soong‐Geun Je, Byoung‐Chul Min, Sonny H. Rhim, Sug‐Bong Choe

**Affiliations:** ^1^ Center for Spintronics Korea Institute of Science and Technology (KIST) Seoul 02792 Republic of Korea; ^2^ Samsung Advanced Institute of Technology (SAIT) Suwon 16678 Republic of Korea; ^3^ Department of Physics University of Ulsan Ulsan 44610 Republic of Korea; ^4^ Materials Science Lab, Department of Physics Quaid‐I‐Azam University Islamabad 45320 Pakistan; ^5^ Department of Physics and Astronomy and Institute of Applied Physics Seoul National University Seoul 08826 Republic of Korea; ^6^ Department of Physics Chonnam National University Gwangju 61186 Republic of Korea

**Keywords:** magnetic domain‐walls, spin polarization, spin‐transfer torques, spintronics

## Abstract

Spin‐transfer torque (STT) and spin‐orbit torque (SOT) form the core of spintronics, allowing for the control of magnetization through electric currents. While the sign of SOT can be manipulated through material and structural engineering, it is conventionally understood that STT lacks a degree of freedom in its sign. However, this study presents the first demonstration of manipulating the STT sign by engineering heavy metals adjacent to magnetic materials in magnetic heterostructures. Spin torques are quantified through magnetic domain‐wall speed measurements, and subsequently, both STT and SOT are systematically extracted from these measurements. The results unequivocally show that the sign of STT can be either positive or negative, depending on the materials adjacent to the magnetic layers. Specifically, Pd/Co/Pd films exhibit positive STT, while Pt/Co/Pt films manifest negative STT. First‐principle calculations further confirm that the sign reversal of STT originates from the sign reversal of spin polarization of conduction electrons.

## Graphical abstract

The sign reversal of spin‐transfer torque (STT) is demonstrated through the magnetic domain‐wall (DW) motion. In Pt/Co/Pt and Pd/Co/Pd magnetic films, STT drives the DW along the current direction and electron‐flow direction, respectively. First‐principles calculations verifies that the reversal of spin polarization of conduction electrons is responsible for this STT sign reversal.

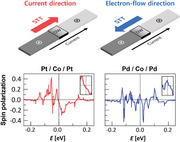

## 1 Introduction

Spintronics facilitates the manipulation of magnets through electric currents. The spin‐polarized electric current, known as the spin current, exerts torque on magnetization,^[^ ^1^, ^2^ ^]^ enabling magnetization reversal^[^ ^3^, ^4^, ^5^ ^]^ or magnetic domain‐wall (DW) motion^[^ ^6^, ^7^ ^]^—fundamental operational principles of magnetic memory,^[^ ^8^, ^9^, ^10^ ^]^ and logic devices.^[^ ^11^, ^12^, ^13^, ^14^ ^]^ Spin‐orbit torque (SOT)^[^ ^15^, ^16^, ^17^ ^]^ and spin‐transfer torque (STT)^[^ ^2^ ^]^ serve as the primary driving forces behind magnetic DW motion, with the key distinction lying in how the spin current is generated. In the case of SOT, the spin current emerges within heavy metals adjacent to magnets, attributed to the spin‐Hall effect^[^ ^15^ ^]^ or interfacial Rashba effect.^[^ ^16^ ^]^ Conversely, in the case of STT, the spin current is generated within magnets, arising from the exchange interaction between the spin of conduction electrons and local magnetization.^[^ ^2^ ^]^

This fundamental difference gives rise to distinct features in SOT‐ and STT‐induced DW motion, particularly in terms of the degree of freedom in its direction. The former has degree of freedom in its direction, while the latter does not. SOT can drive the DW along either the current direction or opposite to the current direction (electron‐flow direction), depending on the combination of the spin‐Hall angle and Dzyaloshinskii–Moriya interaction (DMI), manipulable through material and structural engineering.^[^ ^18^, ^19^, ^20^, ^21^, ^22^, ^23^ ^]^ In contrast, conventional STT theory posits that STT always propels the DW along the electron‐flow direction due to the angular momentum transfer occurring within the conduction electrons within magnets.^[^ ^1^, ^2^ ^]^

However, recent studies by two independent groups challenge conventional STT theories, suggesting the possibility of STT‐induced DW motion along the current direction instead of the electron‐flow direction. Both groups systematically decomposed SOT and STT from the total spin torques exerted on magnetic DWs in Pt/Co/Pt films.^[^ ^24^, ^25^ ^]^ The experimental results revealed that negative STT itself drives the DW along the current direction. Note that if the direction of STT‐induced DW is along the current direction (electron‐flow direction), we refer to it as negative (positive) STT. It is speculated that such negative STT might be attributed either to negative non‐adiabaticity or negative spin polarization of conduction electrons.^[^ ^24^ ^]^ However, neither an engineering scheme for manipulating the STT sign nor verification of the underlying physics of the sign reversal of STT remains known.

In our work, we present the first demonstration of manipulating the STT sign (either positive or negative) by engineering heavy metal layers adjacent to magnetic layers. After measuring total spin torques exerting on magnetic DW motion, we extracted both SOT and STT from the total spin torques, leveraging the anti‐symmetric and symmetric nature of SOT and STT with respect to the handedness of chiral DWs. Various magnetic thin films were investigated (for details, please refer to Section SV, Supporting Information), revealing that Pt/Co/Pt films exhibit negative STT, while Pd/Co/Pd films exhibit positive STT. The fundamental origin of the opposite signs of STT is further verified through first‐principles calculations, attributing them to the spin polarization of conduction electrons. Significantly, our work sheds new light on STT by introducing a novel degree of freedom in its manipulation—the sign of STT—while also providing insight into its physical origin.

## 2 Results

### 2.1 Extraction of the STT and the SOT from Total Spin‐Torques

The STT and SOT are each generated by electric currents flowing through the magnetic layer and the adjacent heavy metal layer, respectively. Consequently, individually quantifying them through conventional electric transport measurements proves challenging. In this study, we introduce symmetry analysis to decompose the STT and SOT, capitalizing on their distinct symmetric behaviors in relation to the handedness of chiral DWs (for details, please see Section SVIII, Supporting Information). As indicated in ref. [24] it is well‐known that STT and SOT exhibit symmetric and anti‐symmetric behaviors with respect to the handedness of chiral DWs, respectively. Therefore, by measuring the total spin‐torques and identifying the symmetric (anti‐symmetric) axis, we can extract the STT and SOT individually.

**Figure 1:**
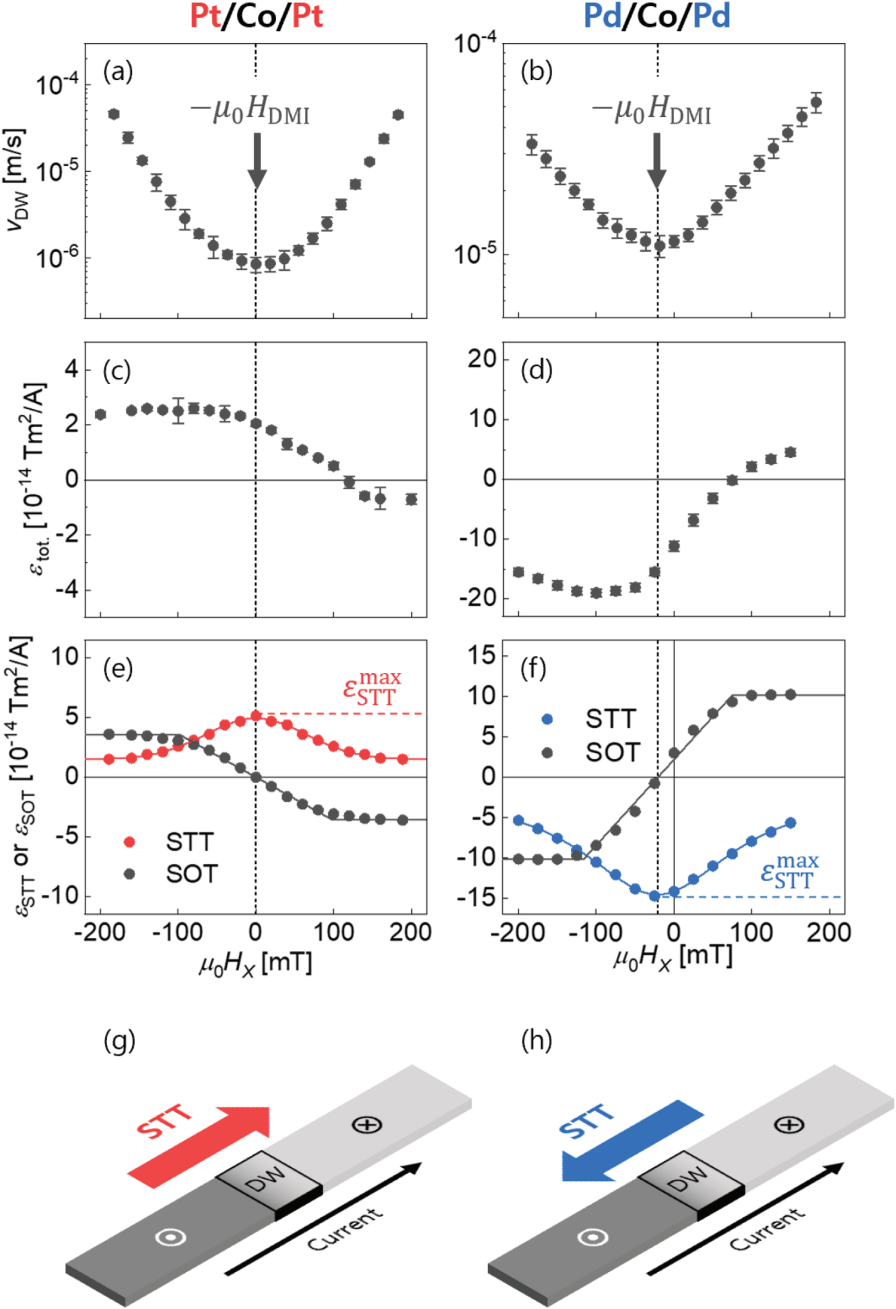
Plots of ***v***_**DW**_ as a function of ***H***_***x***_ for the a) Pt/Co/Pt and b) Pd/Co/Pd films, respectively. The dashed vertical lines indicate ***H***_***x***_ = *** *** − ***H***_**DMI**_. Plots of ***ε***_**tot**._ as a function of ***H***_***x***_ for the c) Pt/Co/Pt and d) Pd/Co/Pd films, respectively. Plots of ***ε***_**STT**_ (colored) and ***ε***_**SOT**_ as functions of ***H***_***x***_ for the e) Pt/Co/Pt and f) Pd/Co/Pd films, respectively. The solid curves guide the symmetric and anti‐symmetric nature of STT and SOT, respectively. The horizontal dashed lines guide eyes to $\varepsilon_{\mathrm{STT}}^{\max}$. Schematic diagrams of STT‐induced DW motion for g) Pt/Co/Pt and h) Pd/Co/Pd films. The red and the blue arrows represent the STT‐induced DW motion along current direction and electron‐flow direction, respectively.

First of all, symmetric/anti‐symmetric axis was determined by measuring the DW speed as a function of in‐plane magnetic field *H_x_*. **Figure** **1a**,b shows the plots of measured *v*_DW_ with respect to *H*_x_ for the Pt/Co/Pt and Pd/Co/Pd films, respectively, where both films have the same *t*_Co_ = 0.3 nm (for detailed film structures, please see Section 4, Experimental Section). According to ref. [26, 27] the symmetric axis (dashed vertical lines) of the *v*_DW_ variation corresponds to the condition *H*_x_ =   − *H*_DMI_, where *H*_x_ exactly compensate *H*_DMI_ so that the chiral DW is of Bloch‐type. This axis will serve as the symmetric/anti‐symmetric axis during the decomposition of STT and SOT from the total spin torques.

The spin‐torques acting on the DW were then measured in these films (for details, please refer to Section SIII, Supporting Information). Following the conventional spin‐torque quantification scheme^[^ ^19^, ^20^, ^21^, ^22^, ^23^ ^]^ we introduce the spin‐torque efficiency ε_tot._, defined as ε_tot._≡ ∂*H*_tot._/∂*j*, where *H*_tot._ is the effective out‐of‐plane magnetic field induced by spin torques, and *j* is the electric current density. By definition, ε_tot._ represents how strongly spin torque is exerted on DWs per unit current density. Figure 1c,d display plots of measured ε_tot._ as a function of *H_x_*. The STT efficiency ε_STT_ and the SOT efficiencyε_SOT_ were then decomposed from ε_tot._ (Please note that ε_tot._ = ε_STT_  + ε_SOT_). As aforementioned, these ε_STT_ and ε_SOT_ are typically symmetric and anti‐symmetric, respectively, for inversion with respect to the axis (dashed vertical lines) of*H*_x_ =  −*H*_DMI_ as given below:

(1) $$\begin{array}{ccc} \varepsilon_{\mathrm{STT}}\left(-H_{x}-H_{\mathrm{DMI}}\right) & = & \varepsilon_{\mathrm{STT}}\left(H_{x}-H_{\mathrm{DMI}}\right)\\ \varepsilon_{\mathrm{SOT}}\left(-H_{x}-H_{\mathrm{DMI}}\right) & = & -\varepsilon_{\mathrm{SOT}}\left(H_{x}-H_{\mathrm{DMI}}\right) \end{array}\;$$

Therefore, one can uniquely decompose the STT and SOT contributions from the experimental variation of ε_tot._(*H_x_*) by the above symmetry argument.

### 2.2 Demonstrating the Sign Reversal of STTs

Figure 1e,f show the results of the decomposition into ε_STT_ (colored) and ε_SOT_^[^ ^28^ ^]^ respectively. The present experimental results deliver two interesting points. First, the signs of STTs are opposite between the Pt/Co/Pt and Pd/Co/Pd films: A positive ε_STT_ is observed in the Pt/Co/Pt film, while a negative ε_STT_ is observed in the Pd/Co/Pd film. The positive ε_STT_ drives DWs along the current direction (so‐called negative STT), while the negative ε_STT_ drives DWs against the current direction (i.e., along the electron‐flow direction). Second, both films exhibit huge ε_STT_ values. Such huge ε_STT_ of Pt/Co/Pt films have been already reported in ref. [26, 27] However, it is interesting to see that the Pd/Co/Pd film exhibits even larger ε_STT_, almost three times larger than the Pt/Co/Pt films, which is the largest value of ferromagnetic film that has been ever reported to the best of our knowledge.

**Figure 2:**
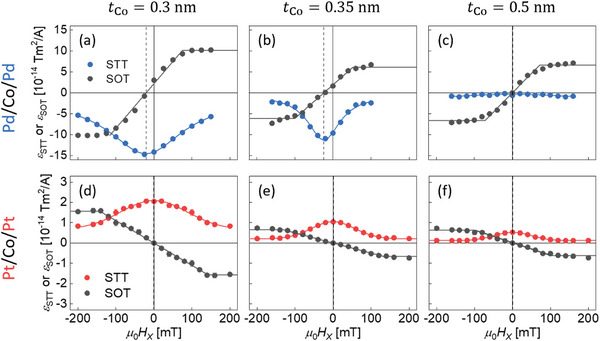
Plots of ***ε***_**STT**_ (colored symbol) and ***ε***_**SOT**_ (black symbol), respectively, as functions of ***H***_**x**_ for the Pd/Co/Pd and Pt/Co/Pt films with different ***t***_**Co**_. The dashed vertical lines indicate ***H***_**x**_ = *** *** − ***H***_**DMI**_. The solid lines show the best fittings of the symmetry and anti‐symmetry of STT and SOT, respectively. The horizontal dashed lines guide eyes to $\varepsilon_{\mathrm{STT}}^{\max}$.

**Figure 3:**
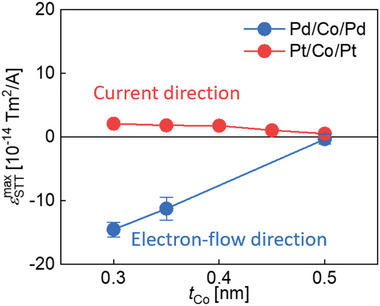
Plots of $\varepsilon_{\mathrm{STT}}^{\max}$ as a function of ***t***_**Co**_ for the Pt/Co/Pt films (red symbol) and Pd/Co/Pd (blue symbol).

To see more detailed nature, the STT was further investigated by changing *t*_Co_ for the series of Pd/Co/Pd and Pt/Co/Pt films. Each panel of **Figure** **2** presents the plot of ε_STT_ (blue for Pd/Co/Pd and red for Pt/Co/Pt) and ε_SOT_ (black), respectively, as functions of *H*_x_ for different *t*_Co_. The results clearly reveal that both of series of Pd/Co/Pd (Pt/Co/Pt) films have positive (negative) ε_STT_ irrespective of *t*_Co_, while the magnitude of ε_STT_ shrinks as increasing *t*_Co_. **Figure** **3** summarizes our STT measurement results by presenting a plot of $\varepsilon_{\mathrm{STT}}^{\max}$ as a function of *t*_Co_ for Pt/Co/Pt and Pd/Co/Pd films. This plot unambiguously demonstrates that one can manipulate not only the sign but also the magnitude of STT through material and structural (thickness) engineering of magnetic films.

According to the STT theories^[^ ^29^ ^]^ ε_STT_ follows the relation ε_STT_ =  (ℏ/2*eM*_S_λ)β*P*, where ℏ is the Planck constant and *e* (> 0) is the absolute value of the elementary charge. Since the saturation magnetization *M*_S_ and DW width λ are also defined as positive quantities, (ℏ/2*eM*_S_λ) is always yields a positive value. Thus, the sign of ε_STT_ follows the sign of β*P*, where β represents the non‐adiabaticity and *P* is the spin polarization of conduction electrons. Recently, Je et al.^[^ ^26^ ^]^ confirmed the appearance of negative β*P* through giant magneto‐resistance measurements in the Pt/Co/Pt films, attributing the negative ε_STT_ in these films to this phenomenon. Both β and *P* generally possess positive values. However, specific situations may arise where either β or *P* becomes negative: DWs narrower than several nanometers^[^ ^30^, ^31^ ^]^ result in a negative β and/or CoPt alloys with dilute Co concentration^[^ ^32^ ^]^ exhibits a negative *P*. Since both β and *P* can change their signs, it remains unclear which one is responsible for the negative sign of STT in these films.

### 2.3 The Microscopic Origin of STT Sign Reversal

To identify the origin responsible for the negative sign of STT, the electronic structure of these films was investigated by first‐principles calculations. A structure of 7‐monolayer Pt (or Pd)/1‐monolayer Co/7‐monolayer Pt (or Pd) is taken into account with *fcc* (111) crystalline lattice (to see XRD spectrum, please find Section SIV, Supporting Information). Then, *P* is estimated as a function of energy *E* by use of the relation $P\left(E\right)\;=\;\frac{g_{↑}\left(E\right)-g_{↓}\left(E\right)}{g_{↑}\left(E\right)+g_{↓}\left(E\right)}\;$ with $g_{\sigma }\left(E\right)\;=\left[\left(1-f_{\mathrm{FD}}\left(E\right)\right)N_{\sigma }^{d}\right]\times N_{\sigma }^{sp}f_{\mathrm{FD}}\left(E\right)$, where $N_{\sigma }^{l}$ is the partial density of state (DOS) for the spin σ (↑ or ↓) and orbital *l* (*s*, *p*, or *d*). Here, *f*_FD_(*E*) is the Fermi–Dirac function that accounts for the occupied *sp* and empty *d* states. Accordingly, a positive (or negative) spin polarization appears when the majority (or minority) spin channel contributes larger than the other. In this simplistic picture, the interaction is accounted as the scattering events between the itinerant *sp* and rather localized *d* electrons.

**Figure 4:**
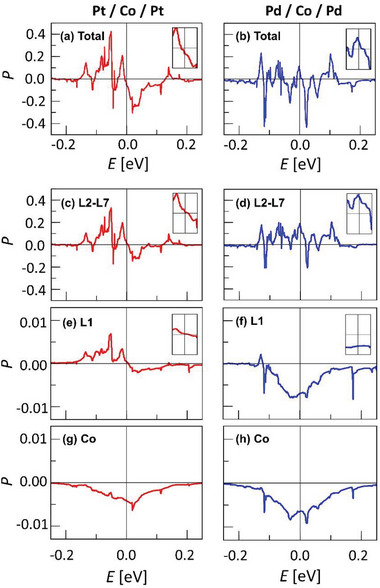
The total ***P*** as function of ***E*** for the a) Pt/Co/Pt and b) Pd/Co/Pd structures, respectively, where ***E***_F_ is set to zero. The layer‐resolved spin polarization contributions: (c) and (d) for the sum of the **L**2 − **L**7 layers, (e) and (f) for the **L**1 layers, and (g) and (h) for the Co‐layer, respectively, for the Pt/Co/Pt and Pd/Co/Pd structures. The insets show ***P*** in the energy window of ***E***_***F***_ ± 0.02 *** *** eV for better visibility.

The calculation results of the total *P* in the Pt/Co/Pt and Pd/Co/Pd structures are shown by **Figure** **4**a,b, respectively, with respect to *E*, where the Fermi level *E*_F_ is set to zero. It is worthwhile to note that the electrons in the vicinity of *E*_F_ dominate conduction. Remarkably, two structures exhibit opposite signs of *P* near *E*_F_, as shown by the insets that *P* < 0 for the Pt/Co/Pt structure and *P* > 0 for the Pd/Co/Pd structure at *E*_F_. The present observation is accordant to the experimental observation of ε_STT_ < 0 for the Pt/Co/Pt films and ε_STT_ > 0 for the Pd/Co/Pd films, signaling that the opposite signs of *P* are responsible for the opposite STTs in these films. Furthermore, the magnitude of *P* in the Pt/Co/Pt structure is smaller than that in the Pd/Co/Pd structure, which is also in accordance to the experimental observation.^[^ ^33^, ^34^ ^]^

The feature of *P* is investigated more in detail by analyzing the layer‐resolved contributions. Figure 4c,d shows the L2 − L7 layer contributions to *P* in the Pt/Co/Pt and Pd/Co/Pd structures, respectively. Also, Figure 4e,f shows the L1 layer contributions to *P* in the Pt/Co/Pt and Pd/Co/Pd structures, respectively. Finally, Figure 4g,h shows the Co layer contributions to *P* in the Pt/Co/Pt and Pd/Co/Pd structures, respectively. It is clearly seen from the figures that, in both structures, the L2 − L7 layer contributions show large *P* values fluctuating over the range up to ± 0.4, whereas the other layer contributions exhibit smaller variation of *P* within the range of ± 0.01. Therefore, the total *P* over the whole structure is dominated by the L2 − L7 layer contributions. It is interesting to note that, in both structures, the Co layers exhibit *P* < 0 in contrast to the bulk characteristics. Such negative *P* might be a consequence of strong electronic hybridization with heavy metal through the interfaces.

Recalling that the electrons in the vicinity of *E*_F_ dominate conduction, in the Pd/Co/Pd structures, the L2 − L7 layer contribution exhibits a large positive *P* (≈ 0.2) as seen in Figure 4d. Such large L2 − L7 layer contribution overwhelms the other contributions of small negative *P* (≈ −0.01) as seen in Figure 4f,h. Therefore, it is natural to see that the total *P* is determined as positive as seen in Figure 4b. On the other hand, in the Pt/Co/Pt structure, the L2 − L7 layer contribution exhibits *P*≅0 as seen by the inset of Figure 4c. In addition, the L1 layer contribution also vanishes in the vicinity of *E*_F_ as seen by the inset of Figure 4e. Thus, the total *P* is dominated by the small negative *P* from the Co layer contribution as seen in Figure 4a,g. The present calculations thus provide an insight on the origin of the opposite *P* in the Pt/Co/Pt and Pd/Co/Pd films. One can therefore conclude that the L2 − L7 layer contribution plays a decisive role in determination of the total *P*.

**Figure 5:**
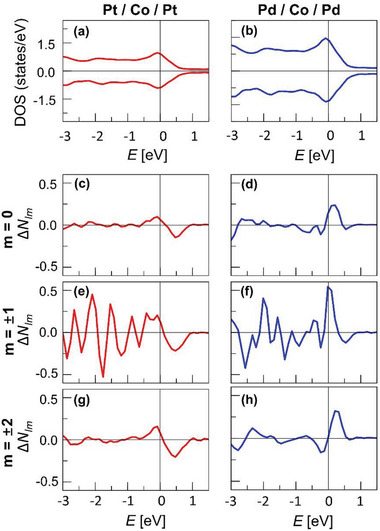
Spin‐dependent DOS of the **L**2 − **L**7 layers as function of ***E*** for the a) Pt/Co/Pt and b) Pd/Co/Pd structures, respectively, where ***E***_F_ is set to zero. Plots of **Δ** ***N***_***lm***_ for ***d*** orbitals with different ***m***: (c) and (d) for ***m *** = *** *** 0, (e) and (f) for ***m *** = *** *** ± 1, and (g) and (h) for ***m *** = *** *** ± 2, for the Pt/Co/Pt and Pd/Co/Pd structures, respectively.

To further elucidate the detailed origin, the DOS of the L2 − L7 layers are shown in **Figure** **5**a,b for the Pt/Co/Pt and Pd/Co/Pd structures, respectively. Although the overall features of the DOS do not differ much, yet the Pd/Co/Pd structure has slightly larger DOS than the Pt/Co/Pt structure in the vicinity of *E*_F_. We further analyze DOS around *E*_F_ in the context of the orbital‐resolved spin polarization. For this analysis, we define $\Delta N_{lm}\;\left(E\right)=N_{lm}^{↑}\;\left(E\right)-N_{lm}^{↓}\left(E\right)$, where $N_{lm}^{\sigma }\left(E\right)$ is DOS at energy *E* for the spin state σ, orbital *l*, and magnetic quantum number *m*. Here, *d* orbitals (*l*  =  2) are decomposed into *m*  =  0, ± 1, and ± 2 based on the irreducible representation of the *fcc* (111) lattice, which are commonly expressed as *z* ^2^ for *m*  =  0, *xz* and *yz* for *m*  =   ± 1, and *x* ^2^ − *y* ^2^ and *xy* for *m*  =   ± 2, respectively. Δ*N_lm_* (*E*) for the Pt/Co/Pt and Pd/Co/Pd structures are plotted: Figure 5c,d for *m*  =  0; in Figure 5e,f for *m*  =   ± 1, and Figure 5g,h for *m*  =   ± 2. Looking close to *E*_F_, one can see that the biggest difference between the Pt/Co/Pt and Pd/Co/Pd structures comes from the case of *m*  =   ± 1, which corresponds to the out‐of‐plane *d* orbitals. Therefore, the out‐of‐plane *d* orbitals play most dominant role in determination of the sign of *P*. The charge density analysis also manifests the dominant role of the out‐of‐plane *d* orbitals (See the Supporting Information).

## 3 Conclusion

We demonstrated the sign reversal of STT through the chiral DW motion, contradicting theoretical predictions. Based on systematic symmetry analysis, we individually quantified both STT and SOT acting on chiral DWs. It was then unambiguously observed that the sign of STT can be reversed depending on the materials adjacent to magnets. In Pd/Co/Pd and Pt/Co/Pt magnetic films, positive and negative STT was exhibited, resulting in DW motion along the electron‐flow and current directions, respectively. First‐principle calculationsverified that the STT sign reversal originates from the spin polarization reversal of conduction electrons within magnets. These findings extend the controllability of STT by introducing a new degree of freedom – its sign – holding promise for emerging spintronic memory/logic devices.

## 4 Experimental Section

### Sample Preparation

For this study, series of Pt/Co/Pt and Pd/Co/Pd films were fabricated on Si/SiO_2_ substrates by DC magnetron sputtering. The detailed stacking structure is 2.5 nm Pt (or Pd)/*t*_Co_ Co/2.5 nm Pt (or Pd) sandwiched by a 5 nm Ta buffer layer and 1.5 nm Pt protection layer. Here,*t*_Co_ = 0.30, 0.35, and 0.50 nm. All the films have strong perpendicular magnetic anisotropy, which is confirmed by square hysteresis loop along out‐of‐plane direction (please see Section SII, Supporting Information).

## Conflict of Interest

The authors declare no conflict of interest.

## Supporting information

Supporting Information

## Data Availability

The data that support the findings of this study are available from the corresponding author upon reasonable request.
