# Supplementary material for: Sign Reversal of Spin‐Transfer Torques
Source: Adv Sci (Weinh). 2024 Apr 16;11(29):2309467. doi: 10.1002/advs.202309467 (PMC11304287; doi:10.1002/advs.202309467)
Supplement: Supplementary file 1 — Supporting Information [file ADVS-11-2309467-s001.pdf]

ADVANCED SCIENCE
Open Access

Supporting Information

for *Adv. Sci.*, DOI 10.1002/advs.202309467

Sign Reversal of Spin-Transfer Torques

Dae-Yun Kim, Qurat ul Ain, Yune-Seok Nam, Ji-Sung Yu, Seong-Hyub Lee, Jun-Young Chang, Kitae Kim, Woo-Young Shim, Duck-Ho Kim, Soong-Geun Je, Byoung-Chul Min, Sonny H. Rhim and Sug-Bong Choe**

Supporting Information

Sign Reversal of Spin-Transfer Torques

Dae-Yun Kim, Qurat ul ain, Yune-Seok Nam, Ji-Sung Yu, Sung-Hyup Lee, June-Yung Chang, Kitae Kim, Woo-Young Shim, Duck-Ho Kim, Soong-Geun Je, Byoung-Chul Min, Sonny H. Rhim, and Sug-Bong Choe**

Contents

Section I. **Curie temperature of magnetic films**

Section II. **Hysteresis loop and magnetic domain image of magnetic films**

Section III. **Determination of spin-torque efficiencies based on domain-wall motion**

Section IV. **XRD spectrum of magnetic films**

Section V. **Magnetic dead layer thickness of various magnetic film structures**

Section VI. **Computational details**

Section VII. **First-principles calculations**

Section VIII. **Influences of STT and SOT on the symmetry of chiral domain-wall speed**

Section IX. **Discussions on SOTs**

Section X. **Vanishing of STTs for thick magnetic layer thickness**

Section XI. **Spin polarization of Co layer**

Section XII. **Effect of roughness on first-principles calculations**

Section XIII. **Estimation of domain-wall width**

Section I. Curie temperature of magnetic films

We determined Curie temperature of magnetic films, by measuring the saturated magnetization as increasing the temperature. Determined Curie temperatures are summarized in the table below. For all magnetic layer thicknesses, Curie temperatures were found to be much higher than the room temperature, which guarantees that present series of magnetic films have strong endurance against to the external thermal fluctuations, such as Joule heating.

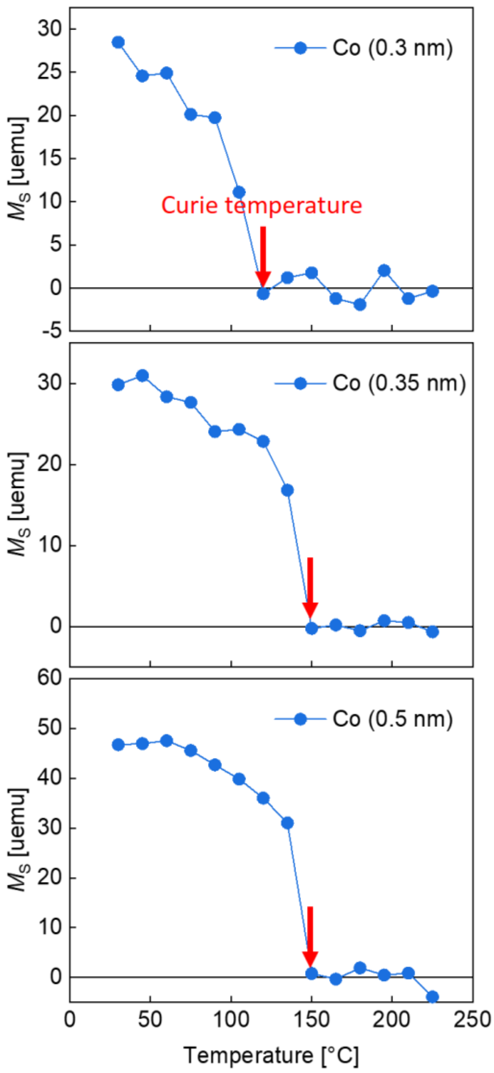

t_{Co} [nm]	T_{Curie} [°C]
0.3	120 ± 10 °C
0.35	150 ± 10 °C
0.5	150 ± 10 °C

Figure S1. Plots of saturation magnetization MS as a function of temperature. Red arrows indicate Curie temperature, where MS becomes zero.

Section II. Hysteresis loop and magnetic domain image of magnetic films

Magnetic hysteresis loops along out-of-plane direction were measured by using vibrating sample magnetometry. Fig. S2 shows hysteresis loop of Pt/Co/Pt and Pd/Co/Pd films. All loops

exhibit square shape, indicating strong perpendicular magnetic anisotropy (PMA). We also observed magnetic domains by using MOKE microscope. Fig. S3 shows normalized magnetic domain images. It is clear that nucleated up domain (black color) circularly propagates, indicating high DW energy density, as well as strong PMA.

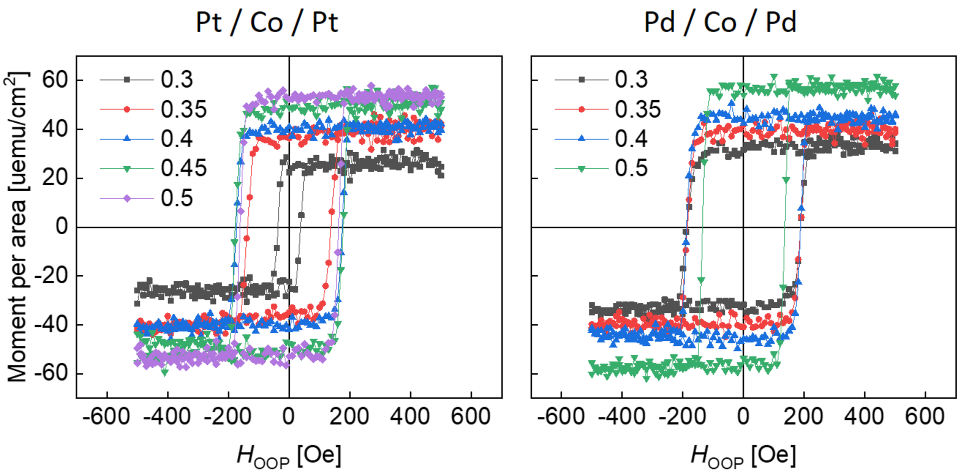

Figrue S2. Easy axis (op-of-plane) loop of present Pt/Co/Pt and Pd/Co/Pd sample series, measured by vibrating sample magnetometer at room temperature.

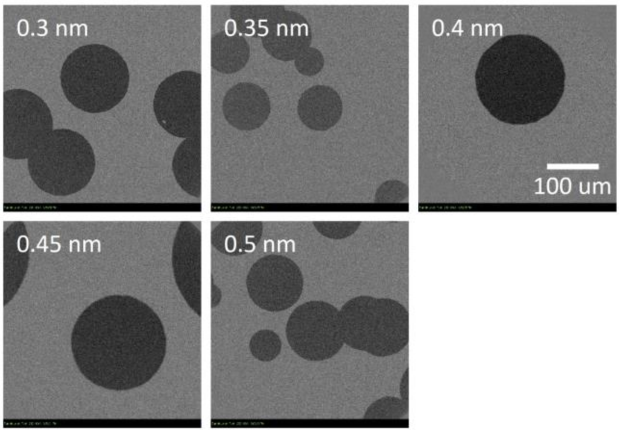

Figure S3. Kerr image of magnetic domains. Each of Dark (black) and bright (grey) area corresponds to up and down domains.

Section III. Determination of spin-torque efficiencies based on domain-wall motion

Spin-torque efficiency was determined by measuring field-driven DW speed with opposite electric current bias $\pm j$. Fig.S4 (a) shows that field-driven DW speed is significantly different

for opposite current bias. This speed difference is solely attributed to the spin-torques generated by electric currents. To quantify the spin-torque, we measured spin-torque-induced effective magnetic field ΔH_{eff}. As shown by Fig.S4 (b), two different curves were shifted along the *x*-axis so that they collapsed into a single curve (dashed line). The magnitude of shift along *x*-axis is the direct measurement of ΔH_{eff}. Then, the spin-torque efficiency $\varepsilon_{\mathrm{ST}}$ is finally determined from $\Delta H_{\mathrm{eff}}/2j$. Figure S5 clearly demonstrates that ΔH_{eff} is linearly proportional to j within the current regime, and thus one can unambiguously determine $\varepsilon_{\mathrm{ST}}$ via best linear fitting.

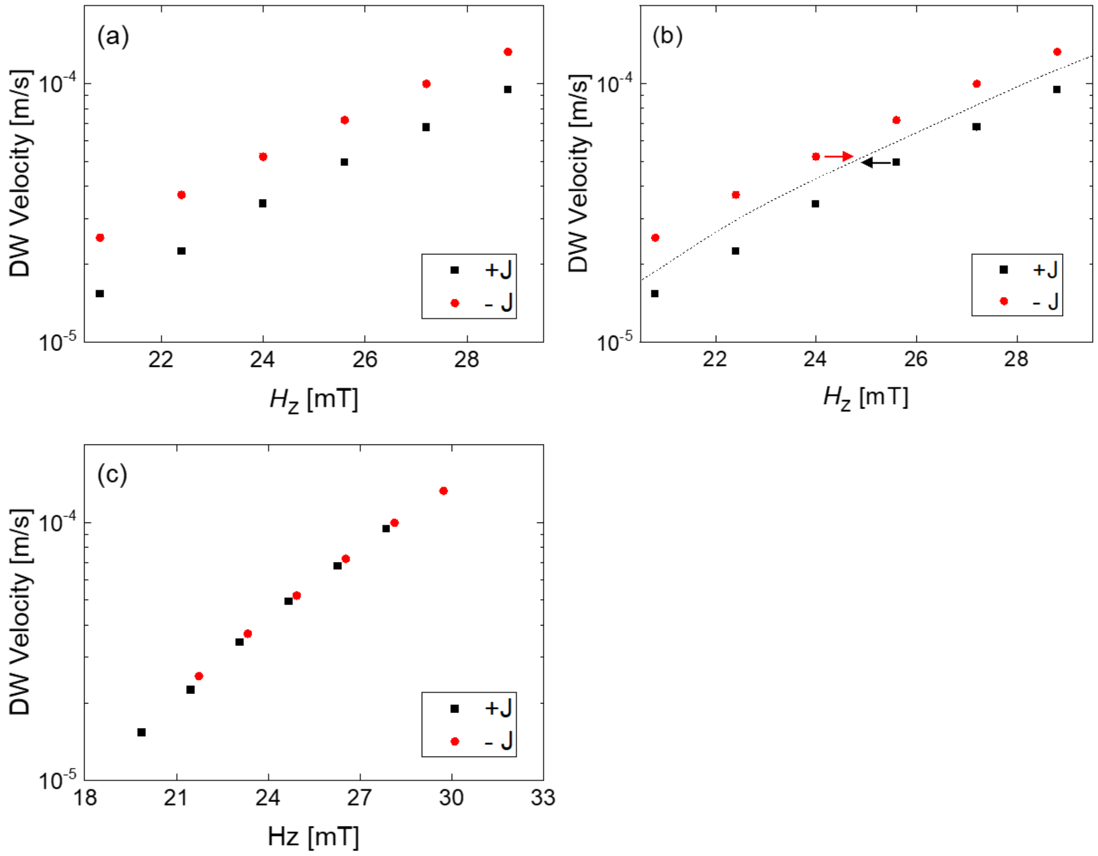

Figure S4. (a) Plots of DW speed as a function of out-of-plane magnetic field H_z. under the application of electric current bias of $+j$ (black) and $-j$ (red). (b) Shifting two curves so that they overlap with each other (dashed curve). Red and black arrows indicate the shift along *x*-axis with amount of ΔH_{eff}. (c) Best interpolation results.

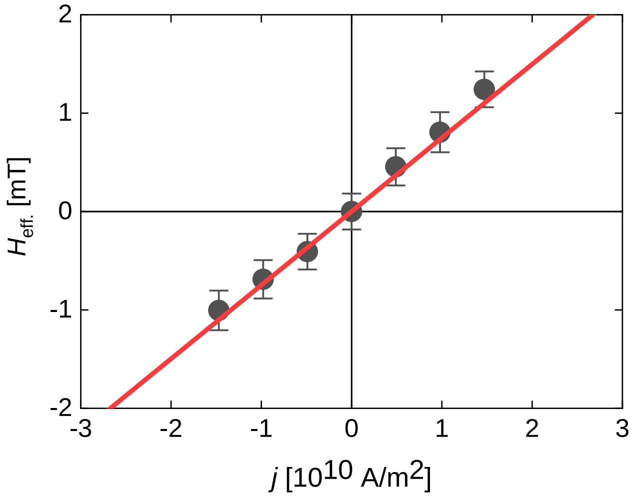

Figure S5. Plot of ΔH_{eff} as a function of j. The red line indicates the best linear fitting with fixed intercept (origin).

Section IV. XRD spectrum of magnetic films

For the experimental part, we measured the X-ray diffraction (XRD) in order to identify the structure of magnetic films. The Fig. S6 shows XRD measurement results. Sharp peak around 39° signifies the feature of fcc 111.

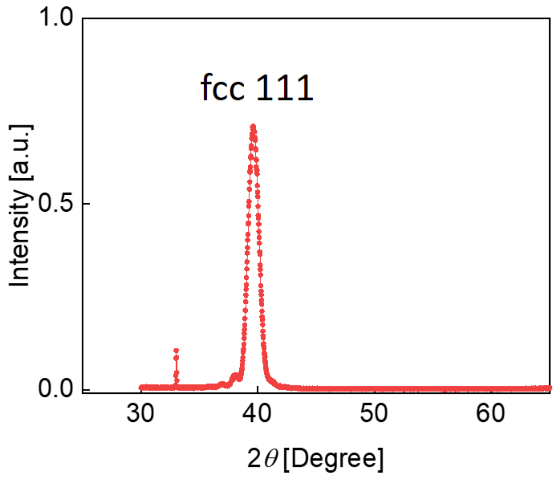

Figure S6. XRD spectrum of 2-theta range of interest

Section V. Magnetic dead layer thickness of various magnetic film structures

To determine magnetic dead layer thickness, we measured magnetic moments per unit area, which corresponds to $M_S t_{Co}$. Fig. S7 shows plots of $M_S t_{Co}$ as a function of t_{Co}. The *x*-intercept of the best linear fitting is the direct measurement of magnetic dead layer thickness t_{Dead}. Both of Pt/Co/Pt and Pd/Co/Pd series of films exhibit ultra-thin t_{Dead} (<0.1 nm).

For the comparison, we also performed identical measurements on other magnetic film structures, such as Pt/Co/Ta, Pt/Co/Ti, and Pt/Co/Ru. As shown in Fig. S8(a), t_{Dead} of these film structures is around 0.5 nm, which is much thicker than that of Pt/Co/Pt and Pd/Co/Pd films. Direct comparison of t_{Dead} values is represented by Fig. S8(b).

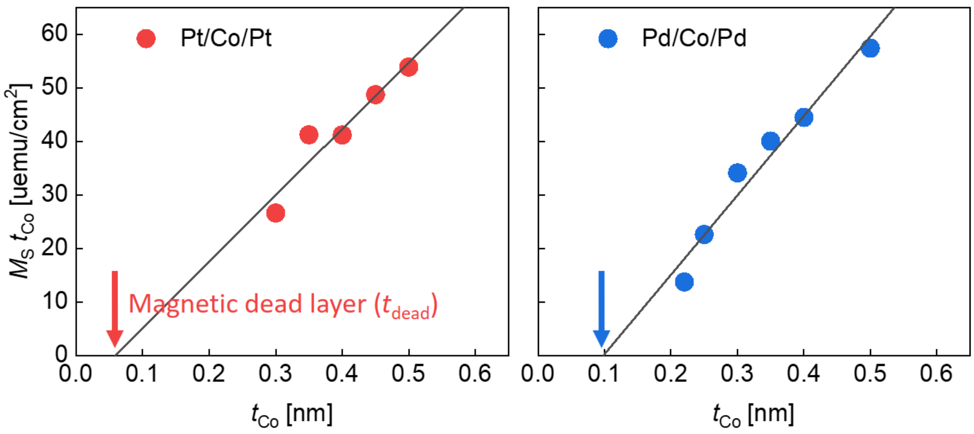

Figure S7. Plots of $M_S t_{Co}$ as a function of t_{Co} for Pt/Co/Pt (red) and Pd/Co/Pd (blue) films. Solid lines are best linear fitting curves. Vertical arrows indicate magnetic dead layer thickness.

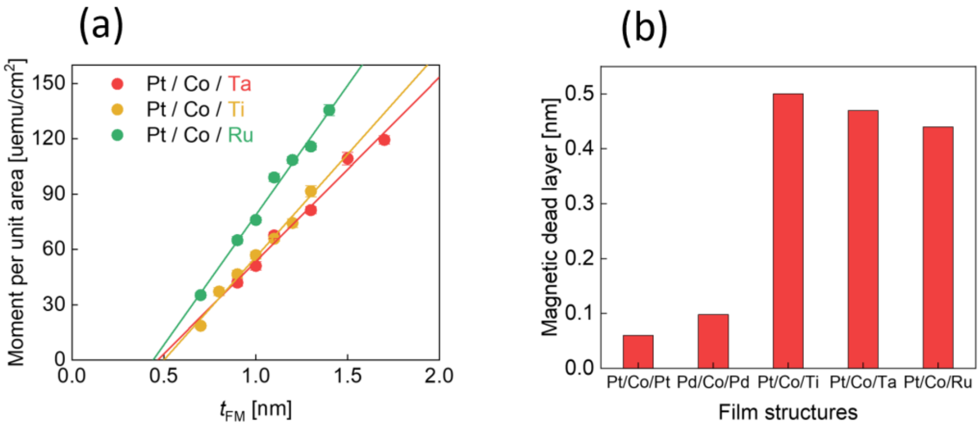

Figure S8. Plots of $M_S t_{Co}$ as a function of t_{Co} for various magnetic film structures. (b) Direct comparison of magnetic dead layer thickness.

Section VI. Computational details

First-principles calculations have been carried out in the framework of the density functional theory as implemented in Vienna *ab initio* simulation package [S1]. Wave functions are described in the basis of the projector-augmented plane wave [S2], where the exchange-correlation potential is obtained via the generalized gradient approximation as parameterized by Perdew-Burke-Ernzerhof [S3]. A k-point grid of 15×15×1 and a plane wave cutoff of 450 eV are used.

Both the Pt/Co/Pt and Pd/Co/Pd structures consist of 1-monolayer of Co sandwiched between 7-monolayers of Pt and Pd, respectively (See the Supplemental Materials). The vacuum region of 1.2 nm is taken between adjacent cells. Layers closest to further from the Co layer are denoted as Ln ($n = 1 \sim 7$). The in-plane lattice constants are adapted from experiment as 0.390 nm and 0.386 nm for the Pt and Pd layers, respectively. The inter-layer distances are relaxed with force criteria 1×10^{-3} eV/Å.

Section VII. First-principles calculations

First-principles calculations are performed with geometry shown in Fig. S9, where Co layer is sandwiched between Pd or Pt seven layers with stacking order of (111) of face-centered cubic lattice. Pd or Pt layers are distinguished by I for interface with Co layer, and I+n for n-the above or below the interface Pd or Pt layer.

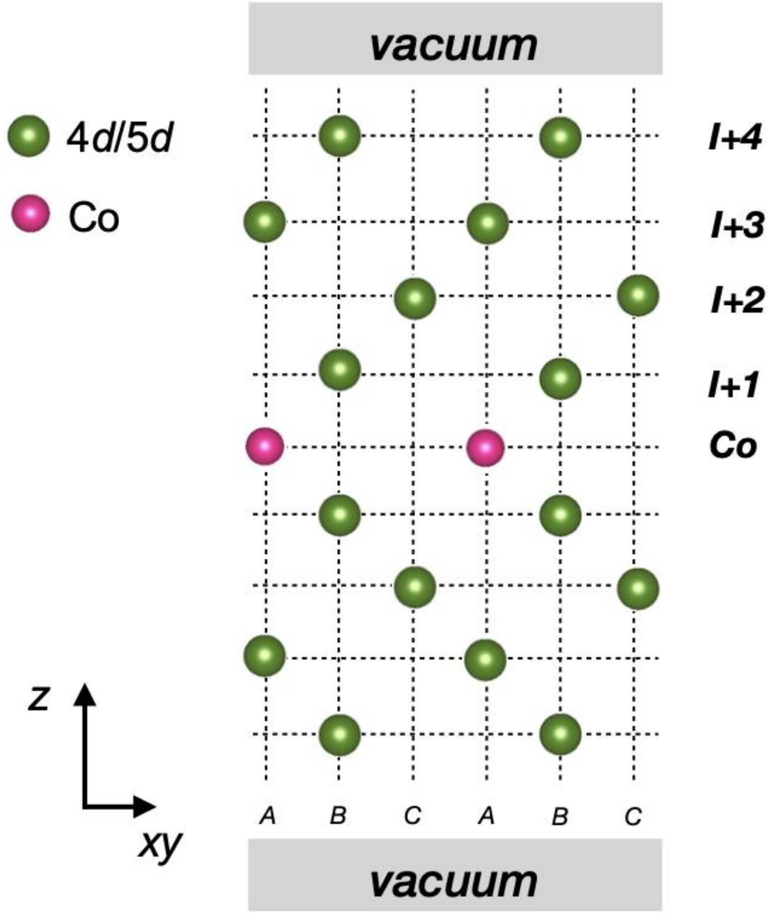

Figure S9: Geometry used in *ab initio* calculations. Co atom in pink sphere and Pd (Pd) are in green spheres. Pd (Pt) layers are denoted *I* for interface, *I+1* for one above or below interface, and so forth. A, B, and C denotes hexagonal sites of the (111) face-centered cubic, which is shown twice in-plane lattice for better visibility.

Table S1. Interlayer distances (in Å) of Pd/Co/Pd and Pt/Co/Pt.

Pd/Co/Pd	Interlayer distance (Å)	Pt/Co/Pt

2.31	(I+5)-(I+6)	2.27
2.31	(I+4)-(I+5)	2.27
2.32	(I+3)-(I+4)	2.27
2.33	(I+2)-(I+3)	2.32
2.34	(I+1)-(I+2)	2.34
2.35	(I)-(I+1)	2.36
2.07	Co-(I)	2.04

Table S2. Magnetic moments (in μ_B) of each layer in Pd/Co/Pd and Pt/Co/Pt.

Pd/Co/Pd	Magnetic Moments (μ_B)	Pt/Co/Pt
0.11	I+2	0.05
0.18	I+1	0.12
0.32	I	0.34
2.0	Co	1.96

The experimental lattice constant of bulk is used for in-plane lattice constant: 0.386 nm for Pd and 0.390 nm for Pt. Considerable lattice mismatch with Co results in different interlayer distances, which are tabulated in Table I. With the presence of Co layer, inward shifts are found by 5-10%. Magnetic moment of each layer is tabulated in Table II. Due to the geometrical setup used in our calculations, moments of (I-n) layers are the same as (I+n) layers. For Pd/Co/Pd structure, magnetic moment of Co layer is 2.0 μB. Pd (I) layer, adjacent to Co, has moment of 0.32 μB, while Pd (I+1) and (I+2) show moments of 0.18 and 0.11 μB, respectively. For

Pt/Co/Pt, on the other hand, Co layer exhibit magnetic moment of 1.96 μB. Pt (I) has induced moment of 0.34 μB. Pt (I+1) shows moment of 0.12 μB and that of Pt (I+2) is negligible.

Section VIII. Influences of STT and SOT on the symmetry of chiral domain-wall speed

Figure S10 illustrates how both STT and SOT impact the symmetry of domain wall speed concerning the chirality of the domain wall. It intuitively depicts how each torque type results in symmetric or anti-symmetric effects on domain walls based on their chirality.

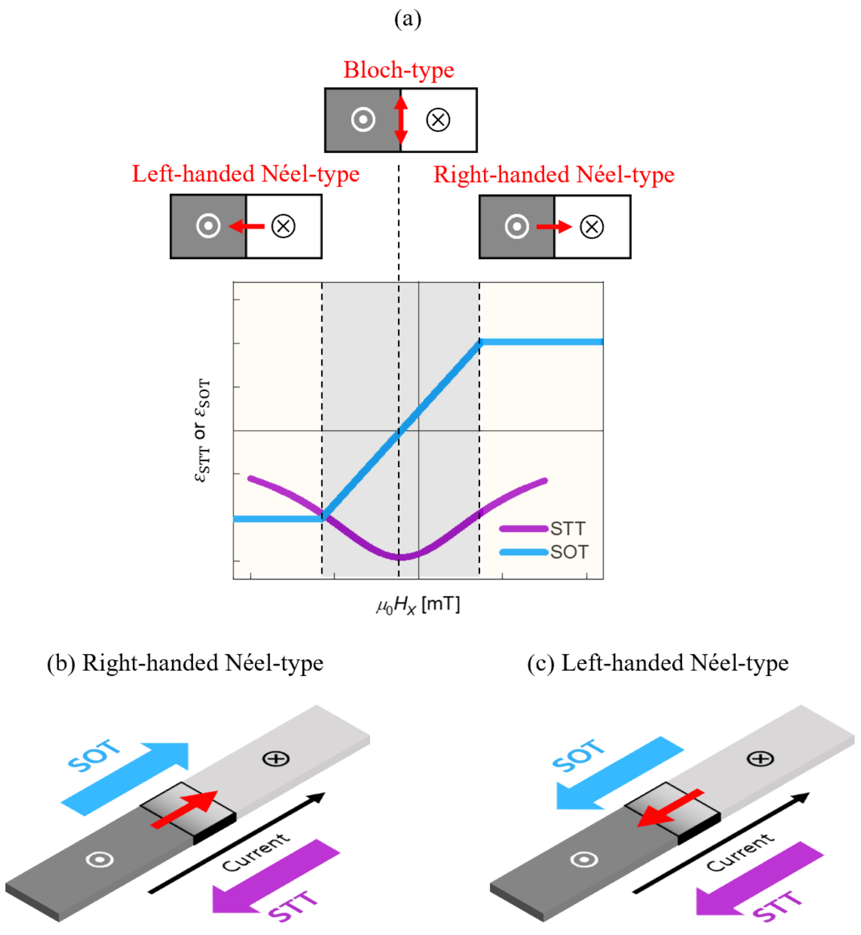

Figure S10. (a) Plot of ε_{STT} and ε_{SOT} as a function of H_x. Red arrow represents the direction of DW center magnetization (i.e. DW chirality). Schematic illustration of STT and SOT acting on DW with (b) right-handed Néel-type and (c) left-handed Néel-type chirality. Sky blue and purple arrows indicate the direction of SOT and STT.

Section IX. Discussions on SOTs

The sign of SOT acting on domain walls is determined by the sign of the total spin current injected from the heavy metal layers induced by the spin-Hall effect (SHE). As noted by the referee, Pt/Co/Pt and Pd/Co/Pd films exhibit opposite SOT signs. This indicates that the sign of the total spin current injected from adjacent heavy metal layers for these two film structures is opposite. It is natural to speculate that the SOT sign of Pt/Co/Pt films is primarily influenced by the spin current injection from the upper heavy metal layers, given that the upper Pt layer is thicker than the lower Pt layer, and the bottom Ta layer has an opposite spin Hall angle with respect to Pt. Consequently, it can be inferred that the SOT sign of Pd/Co/Pd films is determined by the lower heavy metal layers. However, determining the exact origin of the opposite SOT signs between these two film structures is challenging due to factors such as interface roughness, spin Hall conductivity, and others, which also influence the SOT efficiency of current-induced domain wall motion.

Section X. Vanishing of STTs for thick magnetic layer thickness

Figure S11 illustrates plots of $\varepsilon_{\mathrm{tot.}}$ as a function of H_x for Pd/Co/Pd films with thicker Co layer thickness (0.6, 0.8 nm). Notably, it is evident from this figure that $\varepsilon_{\mathrm{tot.}}$ exhibits anti-symmetric behavior with respect to $H_x = 0$, indicating that the total spin-torque is primarily attributed to the SOT, while the contribution from STT is almost negligible (i.e. $\varepsilon_{\mathrm{STT}} \approx 0$). It's important to recall that $\varepsilon_{\mathrm{tot.}} = \varepsilon_{\mathrm{STT}} + \varepsilon_{\mathrm{SOT}}$, where each component, $\varepsilon_{\mathrm{STT}}$ and $\varepsilon_{\mathrm{SOT}}$, demonstrates symmetric and anti-symmetric behavior with respect to H_x.

As shown by Figure S12, this unexpected finding challenges conventional expectations, as one might naturally anticipate a larger and positive STT contribution with increasing Co layer thickness due to the expected bulk contribution of the magnetic layers. However, our

observation indicates a contrary trend where the STT efficiency diminishes with thicker Co layers. This unexpected behavior can be attributed to the inverse relationship between STT efficiency and domain-wall (DW) width (i.e. $\varepsilon_{\mathrm{STT}} \propto \lambda^{-1}$). As the Co layer thickness increases, the DW width also expands rapidly, leading to a significant reduction in the STT efficiency, making it almost negligible compared to the SOT (please find Y.-S. Nam *et. al.*, Applied Physics Letters **121**, 072403 (2022)). In addition, we would like to reference our group's previous experimental results (please see Y.-K. Park *et al.*, NPG Asia Mater. 10, 995 (2018)). In this study, spin-torques were measured for Pt(2.5)/Co(0.9, much thicker than our case)/X(2.5)/Pt(1.5 nm) films, where X=Pt, Pd, W, Ta, Ru, Ti, Al, Au, Cu. Despite changing the upper layer materials with nine different materials, none of them exhibited STT. We believe that these experiments support our conclusion that STT vanishes when the Co layer is sufficiently thick enough (~0.9 nm).

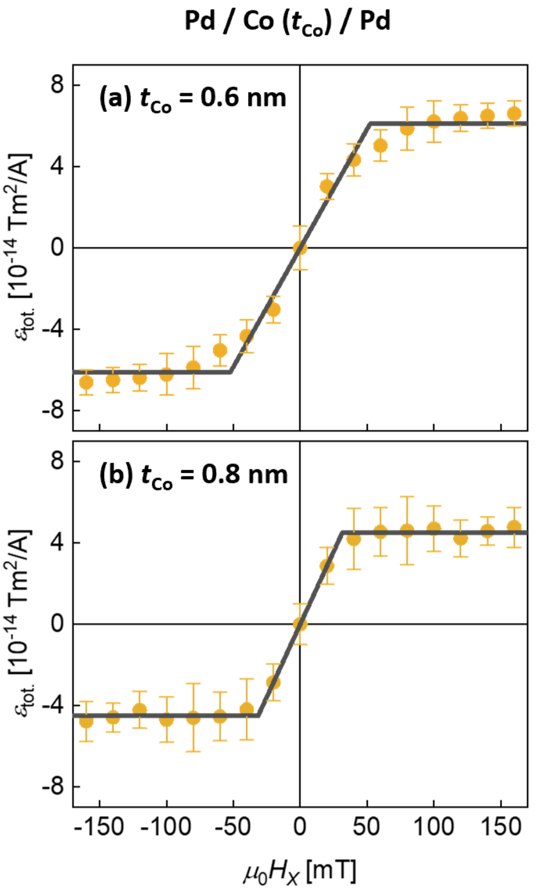

Figure S11. Plots of $\varepsilon_{tot.}$ as a function of $\mu_0 H_x$ for Pd/Co/Pd films with thick Co layer thickness.

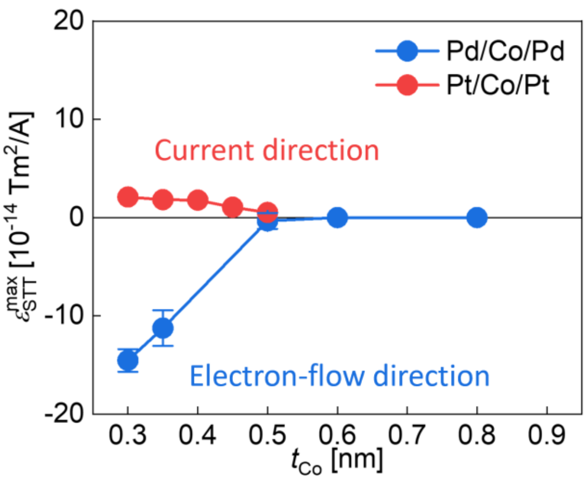

Figure S12. Plots of ε_{STT}^{max} as a function of t_{Co} for Pt/Co/Pt (red) and Pd/Co/Pd (blue) films.

Section XI. Spin polarization of ultra-thin Co layers

Widely known P values of Co are 0.3~0.5, which however are measured in multilayer with thickness of 10~20 nm. When Co gets thinner approaching one monolayer, P approached to 0.1 which agrees quite well with our estimate.

Works reporting P around 0.5 are:

- Rajanikant et al, Appl. Phys. Lett. **97**, 022505 (2010): P ~ 0.56 for [Co/Pt] with 15 nm
- N. Satchell et al, Sci. Rep.13, 12468 (2023).: P ~ 0.47 for [Co50P50](128nm) /Pt(4nm)

Works reporting P around 0.1 are:

- F. Meier et al. Phys. Rev. B, **83**, 075407 (2011): P ~ 0.1 for Co nanostrip on Pt (111)
- O. Sipr et al, J. Phys.: Condens. Matter **19**, 096203 (2007): P ~ 0.1 for ab initio work on Co adatom on Pt(111)

While exact quantification of P value of Co seems still open, the tendency is clear. The thinner Co thickness, the closer P value to 0.1, which is our case whereas the thicker Co film, the closer P to 0.3~0.5, which referee mentioned.

Section XII. Effect of roughness on first-principles calculations

Before we discuss the effect of roughness on first-principles calculations, one has to define and clarify the degree of roughness associated with inhomogeneity, which depends on sputtering condition. Ignoring complexity by considering only one “well-defined” isolated roughness, one can imagine formation of quantum well geometry. The nature of the quantum well geometry can be parametrized by width and depth. With limited knowledge, one study is by L. Zhou *et al.*, Physical Review B **91**, 045407 (2015), where surface roughness can enhance spin Hall conductivity in Cu and Al comparable to Au even without spin-orbit coupling. In earlier century, roughness effect has been studied with theoretical rigor by Tesanovic *et al*, Phys. Rev. Lett. **57**,

2760 (1986), which has shown that qualitatively different transport phenomena from quasi-classical regime can emerge. However, the work also pointed out that such qualitative change depends on impurity, degree of roughness and etc.

Section XIII. Estimation of domain-wall width

Following the analysis method suggested by Y.-S. Nam et. al., Applied Physics Letters **121**, 072403 (2022), we calculated the DW width values using the H_S and M_S values for our samples, where H_S represents the DW anisotropy field and M_S indicates the saturation magnetization measured from VSM. It's important to note that H_S is defined as the external magnetic field needed to transition the DW chirality from Bloch-type to Néel-type, which can be readily extracted from Figure 2 of the main text. Figure R5, a copy of Figure S13 from the revised Supporting Information, presents the plot of DW width as a function of Co layer thickness for Pt/Co/Pt and Pd/Co/Pd films. As depicted in the figure, the DW width is estimated to be even narrower than a nanometer for both Pt/Co/Pt and Pd/Co/Pd films. According to Ref. 30 in the revised main text, the non-adiabaticity parameter β can be negative in this regime of DW width. However, given that both Pt/Co/Pt and Pd/Co/Pd films exhibit sub-nanometer DW width, it is challenging to determine whether the sign reversal of STT between the two films is attributed to the sign reversal of β or not.

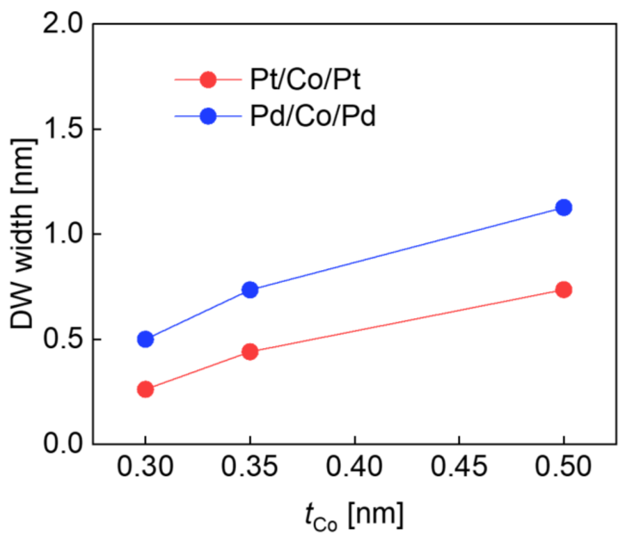

Figure S13. Plot of estimated DW width as a function of Co layer thickness for Pt/Co/Pt and Pd/Co/Pd films.

References

[S1] G. Kresse, and J. Furthmüller, Phys. Rev. B **54**, 11169 (1996).

[S2] P. E. Blöchl, Phys. Rev. B 50, 17953 (1994).

[S3] J. P. Perdew, K. Burke, and M. Ernzerhof, Phys. Rev. Lett. **77**, 3865 (1996).
